# High prevalence of antinuclear antibodies in patients with chronic hepatitis C virus infection

**DOI:** 10.1186/s40001-022-00809-6

**Published:** 2022-09-16

**Authors:** Geison Luiz Costa de Castro, Ednelza da Silva Graça Amoras, Mauro Sérgio Araújo, Simone Regina Souza da Silva Conde, Carlos David Araújo Bichara, Maria Alice Freitas Queiroz, Antonio Carlos Rosário Vallinoto

**Affiliations:** 1 Virology Laboratory, Institute of Biological Sciences, Federal University of Pará, Belém, Pará Brazil; 2 School of Medicine, Federal University of Pará, Health Science Institute, Belém, Pará Brazil; 3 ‘João de Barros Barreto’ University Hospital, Federal University of Pará, Belém, Pará Brazil; 4 ‘Amaral Costa’ Laboratory of Diagnostic Medicine, Belém, Pará Brazil; 5 Postgraduate Program in Infectious Agent and Parasite Biology, Federal University of Pará, Belém, Pará Brazil

**Keywords:** HCV, ANA, Genotypes, Autoantibodies

## Abstract

**Background:**

Hepatitis C virus (HCV) infection is a serious public health concern due to its high prevalence and mortality rate. In chronic infection, HCV may induce autoimmune responses through the production of autoantibodies, including antinuclear antibodies (ANA).

**Methods:**

We assessed the presence of ANA by indirect immunofluorescence using HEp-2 cells in 89 patients with chronic hepatitis C. We also collected data on epidemiological variables; clinical characteristics; and biochemical, hematological, molecular, and histopathological information from the patients to assess the impact of the presence of ANA in those patients.

**Results:**

The prevalence of ANA in the patients was 20.2%, which was significantly higher than that found in healthy controls (2%). However, there was no association of this marker with epidemiological, clinical-laboratory, molecular or histopathological characteristics of hepatitis C, although a slightly higher prevalence of ANA was detected in women and in patients infected with subgenotype 1a. In a specific analysis, chronic HCV patients with the “rods and rings” cytoplasmic pattern had higher degrees of hepatic fibrosis than did ANA-negative patients.

**Conclusions:**

The results confirm a greater predisposition to the presence of ANA in patients with HCV, which may be associated with a worse prognosis, especially in the presence of the “rods and rings” cytoplasmic pattern.

## Introduction

Hepatitis C virus (HCV) infection has a substantial impact on public health, occurring in its chronic form in about 71 million individuals [1–3]. The disease also leads to the death of more than 400,000 carriers per year due to complications arising from chronic infection, such as liver cirrhosis and hepatocellular carcinoma [4, 5].

In addition to causing liver damage, chronic hepatitis C has been associated with the production of non-organ-specific autoantibodies, such as antinuclear antibodies (ANA) [6]. In fact, several studies have shown the presence of ANA in a significant number of patients with chronic HCV infection, reaching more than 40% of cases [7–9]. However, the mechanisms that link HCV to autoimmune processes are not well established. Although the occurrence of HCV proteins that mimic host molecules has been documented [10, 11], treatment with interferon (IFN) is one of the main factors related to the occurrence of antibodies against pancreatic cells [12] and the occurrence of anti-thyroid autoantibodies in patients with chronic HCV infection [13].

Although several studies have shown significant associations between the presence of ANA and certain clinical and laboratory characteristics of chronic HCV infection, these studies have not shown a pattern that can elucidate its role in infection [8]. In an attempt to determine the relevance of the presence of ANA in patients with chronic HCV infection, the objective of this study was to describe the prevalence of ANA in these individuals and to investigate possible associations between the presence of ANA and clinical-laboratory, molecular, and histopathological characteristics to evaluate the influence of these molecules on the progression of infection and of liver disease.

## Methods

### Study population

The present study was cross-sectional and analytical. The study group consisted of 89 chronic HCV carriers attended at Santa Casa de Misericórdia Foundation of Pará and “João de Barros Barreto” University Hospital of the Federal University of Pará. Sample and patient data collections took place between 2013 and 2016. The inclusion criteria for the patients were chronic HCV carrier, age older than 18 years, viral RNA detectable, not undergoing treatment during the collection period and availability of data on clinical characteristics and liver biochemical tests. The exclusion criteria were age less than 18 years old, coinfection with hepatitis B virus (HBV) or human immunodeficiency virus (HIV), and previous diagnosis of autoimmune hepatitis, since coinfection with these viruses can modulate disease progression [14, 15] and the diagnosis of autoimmune hepatitis could bias in the interpretation of ANA test results [16]. Additionally, a group of 100 blood donors with negative serologies for HCV, HBV, and HIV was used to estimate the prevalence of ANA in the healthy population.

### Biological samples

Patients’ blood samples were collected by venipuncture. Liver biopsy samples were obtained from patients after medical indication for evaluation of possible abnormalities in the liver parenchyma. Specimens were collected using an ultrasound-guided Trucut needle biopsy procedure. Liver biopsy specimens were examined at the Department of Anatomical Pathology, Federal University of Pará, using the METAVIR scoring system for histopathological evaluation [17].

### Complementary tests

The detection of viral RNA, determination of HCV genotype and subgenotype, as well as viral load (VL) were carried out at the Central Laboratory of the State of Pará (LACEN). Detection of viral RNA and determination of viral load were performed using the RT-PCR method (AMPLICOR MONITOR^®^, Roche Molecular Systems). In 78 patients the viral genotype and subgenotype were determined by sequencing the 5′ untranslated region of HCV using the Linear Array Hepatitis C Virus Genotyping Test (LiPA-Line Probe Assay-Roche Diagnostics).

The evaluation of biochemical markers was performed in the clinical analysis laboratories of both hospitals. Tests included the quantification of alanine aminotransferase (ALT), aspartate aminotransferase (AST), gamma-glutamyl transferase (GGT), alkaline phosphatase (ALP), total bilirubin (TB), direct bilirubin (DB) and indirect bilirubin (IB), total protein (TP), and fractions (albumin and globulin); determination of the platelet count; and evaluation of the prothrombin time (PT). Complementary tests also included abdominal ultrasound and esophagogastroduodenoscopy.

### Analysis of antinuclear antibodies

ANA screening procedures were performed at the Virology Laboratory of the Federal University of Pará on all plasma samples from the group of patients with chronic HCV infection and from the group of healthy controls. An ANA qualitative evaluation was performed using indirect immunofluorescence (IIF) with a VIRGO Antinuclear Antibody/ANA/Hep-2 kit (Hemagen Diagnostics, USA) according to the manufacturer’s instructions. Reactive samples were those that showed reactivity at 1/80 titration, as recommended in the IV Brazilian Consensus for Autoantibodies Screening in HEp-2 Cells [18].

### Statistical analysis

BioEstat version 5.4 and SPSS version 22 were used for statistical analysis of the data. The Chi-square test, Fisher's exact test, and the *G* test were used for the analyses of quantitative parameters. First, the Kolmogorov–Smirnov test was performed to assess the normality of the data. Subsequently, Student’s *t* test, the Mann–Whitney test, ANOVA, and the Kruskal–Wallis test were used. The null hypothesis, referring to the lack of an association between the factors evaluated, was rejected when a *p*-value lower than 0.05 was obtained.

## Results

The mean age of the evaluated patients was 54.4 ± 9.3 years, and 52.8% were male. The majority of patients had genotype 1 virus (74.7%). ANA positivity was detected in 18 patients (20.2%), which represented a statistically significant difference compared with the percentage of positive cases (2%) in the control group (*p* < 0.0001).

In the patient group, cytoplasmic patterns prevailed over nuclear and mixed patterns, and mitotic spindle patterns were not detected. The cytoplasmic “rods and rings” (*RR*) pattern had the highest prevalence, occurring in 11 (61.1%) individuals. The other cytoplasmic patterns detected were the cytoplasmic discrete dot pattern and the cytoplasmic dense fine speckled pattern. The detected nuclear patterns were the nuclear fine speckled pattern, the homogeneous nucleolar pattern, and the nuclear dense fine speckled pattern. A single sample had a mixed pattern: nuclear and fine speckled nucleolar type with a positive metaphase plate and decoration of the cytoplasm and of the nucleolus organizer region (Table 1). In the control group, one individual had a fibrillar cytoplasmic pattern and the other had a polar speckled cytoplasmic pattern.

**Table 1:** Prevalence of ANA patterns detected

Pattern	Occurrence (%)
Nuclear patterns	4 (22.2)
Nucleolar homogeneous	2 (11.1)
Fine speckled	1 (5.6)
Dense fine speckled	1 (5.6)
Cytoplasmic patterns	13 (72.2)
Rods and rings	11 (61.1)
Discrete dot	1 (5.6)
Dense fine speckled	1 (5.6)
Mixed patterns	1 (5.6)
Nuclear and nucleolar fine speckled with positive metaphase plate	1 (5.6)

No significant differences were found in the prevalence of ANA according to factors such as age, sex, alcohol consumption, smoking, drug use, and previous cases of hepatitis (Table 2).

**Table 2:** General data for the evaluated patients

	ANA-positive	ANA-negative	*p-*value
Age	55.55 ± 8.3	53.71 ± 10.77	0.9526^T^
Female sex	9 (50%)	32 (45.08%)	0.9124^Q^
Alcohol consumption	8 (44.44%)	33 (50%)	0.6766^Q^
Smoking	6 (33.33%)	21 (31.34%)	0.8721^Q^
Injectable drugs	2 (12.5%)	4 (6.06%)	0.5908^E^
Inhalant drugs	2 (12.5%)	5 (7.58%)	0.8963^Q^
Previous hepatitis	2 (11.76%)	10 (14.71%)	0.7308^E^

^E^Fisher's exact test; ^Q^Chi-square test; ^T^*T* test

All patients underwent clinical evaluation; endoscopy was performed in 60 (67.41%) and abdominal ultrasounds in 79 (88.76%) of the patients. In this context, systemic arterial hypertension was the most frequent clinical feature in ANA-positive patients, occurring in 8 (44.4%) patients. Liver biopsies were performed in 64 patients (71.91%). When the ANA-positive and ANA-negative patient data were compared, no significant differences were detected in the frequencies of any of the evaluated clinical characteristics or at the histopathological level (Table 3).

**Table 3:** Clinical and histopathological data for the patients evaluated in this study

Clinical characteristic	ANA-positive	ANA-negative	*p-*value
Systemic arterial hypertension	8 (44.4%)	24 (35.8%)	0.1243^Q^
Diabetes mellitus 2	2 (11.1%)	13 (19.4%)	0.5093^E^
Hepatomegaly	0	11 (16.4%)	0.1100^E^
Hepatic steatosis	5 (31.2%)	11 (17.2%)	0.1278^Q^
Cirrhosis	6 (33.3%)	9 (12.7%)	0.0708^Q^
Necroinflammatory activity^G^			
A0	1 (8.33%)	4 (7.55%)	0.6820
A1	7 (58.33%)	27 (50.94%)	
A2	4 (33.3%)	18 (33.96%)	
A3	0	4 (7.55%)	
Degree of fibrosis^G^			
F0	0	4 (7.55%)	0.0770
F1	2 (16.67%)	20 (37.73%)	
F2	3 (25%)	16 (30.18%)	
F3	4 (33.3%)	12 (22.64%)	
F4	3 (25%)	1 (1.89%)	

^Q^Chi-square test; ^E^Fisher's exact test; ^G^*G* test

In the analyses of VL and biochemical and hematological markers, there were also no statistically significant differences between the values obtained from ANA-positive and ANA-negative patients (Table 4).

**Table 4:** Quantification of the laboratory markers of the patients evaluated in this study

Marker	ANA-positive	ANA-negative	*p-*value
AST (U/L)	88.8 ± 76.18	65.85 ± 37.88	0.2351^M^
ALT (U/L)	85.75 ± 70.29	77.59 ± 54.47	0.6039^T^
GGT (U/L)	104.87 ± 82.04	95.1 ± 92.95	0.4705^M^
ALP (U/L)	121.98 ± 51.94	119.13 ± 56.18	0.8533^T^
TB (mg/dL)	1.29 ± 1.49	0.86 ± 0.47	0.8300^M^
DB (mg/dL)	0.42 ± 0.44	0.27 ± 0.17	0.9340^M^
IB (mg/dL)	0.92 ± 1.25	0.59 ± 0.38	0.3863^M^
TP (g/dL)	7.4 ± 0.7	7.57 ± 1.01	0.3938^M^
Albumin (g/dL)	4.08 ± 0.54	4.23 ± 0.65	0.4147^T^
Globulin (g/dL)	3.37 ± 0.76	3.42 ± 0.95	0.8344^T^
PT (%)	79.75 ± 17.58	83.32 ± 20.51	0.5536^T^
Platelets (× 10^3^)	173.82 ± 78.6	203.2 ± 76.31	0.1610^T^
VL (log_10_)	5.59 ± 0.35	5.49 ± 0.84	0.8748^M^

^M^Mann–Whitney test; ^T^*T* test

**Fig. 1:**
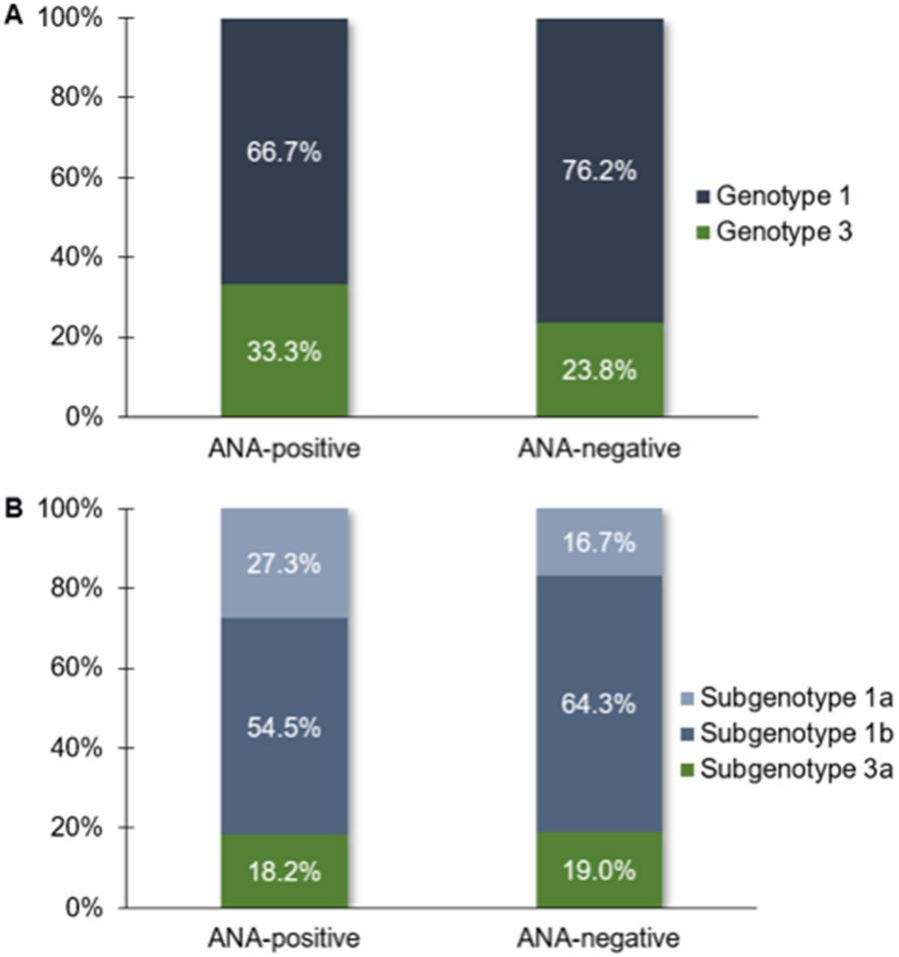
**a** Distribution of HCV genotypes in ANA-positive and ANA-negative patients. **b** Distribution of HCV subgenotypes in the same groups of patients

In the analysis of HCV genotypes (Fig. 1), a higher frequency of patients with genotype 1 and HCV subgenotype 1b was observed in both ANA-positive and ANA-negative patients, such that no significant differences were observed in the genotype (Fisher's exact test, *p* = 0.7485) or subgenotype (*G* test, *p* = 0.5028) distributions.

**Fig. 2:**
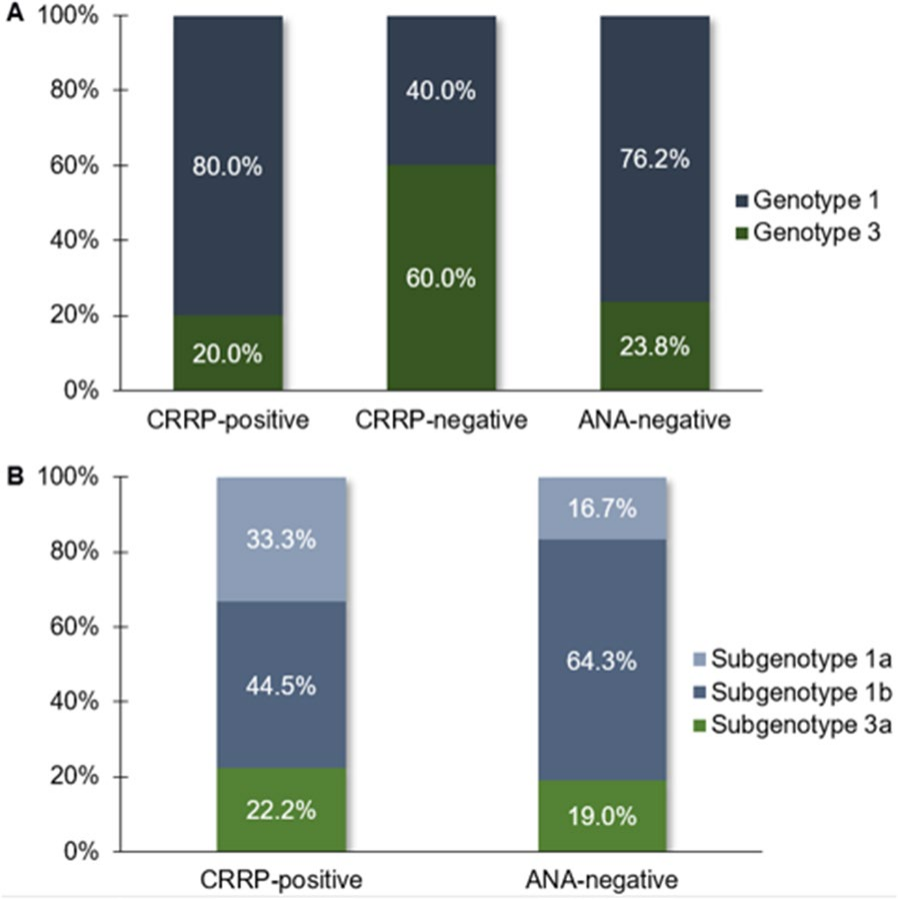
**a** Distribution of HCV genotypes in ANA-positive *RR*-positive, ANA-positive *RR*-negative, and ANA-negative patients. **b** Distribution of HCV subgenotypes in the same groups of patients

Genotype 3 was more frequent in ANA-positive patients. Genotype 1 was more frequent in ANA-positive patients with *RR* and in ANA-negative patients, and genotype 3 was more frequent in ANA-positive patients with other patterns. However, this distribution was not statistically significant between these subgroups (*G* test, *p* = 0.3055). A comparison between frequencies of HCV subgenotypes was performed only using the data relative to individuals from the subgroups of ANA-positive patients with *RR* and ANA-negative patients due to the low number of samples from ANA-positive patients without *RR* who underwent subgenotype analysis. Thus, subgenotype 1b was the most frequent in both subgroups, resulting in the absence of a significant difference in their distribution (*G* test, *p* = 0.1596), although a higher prevalence was observed in patients with subgenotype 1a with *RR* (Fig. 2).

Taking into account the remarkably high prevalence of ANA-positive patients with *RR* (61.1%), the epidemiological, clinical-laboratory, molecular, and histopathological variables of these patients were compared with those of the other ANA-positive patients and with those of ANA-negative patients.

The mean age observed in the subgroup of ANA-positive patients with *RR* was 52.81 ± 9.7 years, with no statistically significant difference when compared to the mean age of the subgroups of patients with other ANA patterns (54.71 ± 5.99) and of ANA-negative patients (53.71 ± 10.77) (*p* = 0.9292). Likewise, no statistically significant difference was observed when analyzing data on the use of injectable drugs, use of inhaled drugs, alcohol consumption, smoking, and prior episode of hepatitis of any etiology (Table 5).

**Table 5:** Comparison of epidemiological data for ANA-positive patients *RR*-positive, ANA-positive *RR*-negative, and ANA-negative patients

	*RR*-positive *N* (%)	*RR*-negative *N* (%)	ANA-negative *N* (%)	*p-*value
Age	52.81 ± 9.7	54.71 ± 5.99	53.71 ± 10.77	0.9292^A^
Female sex	4 (36.36%)	5 (71.42%)	3 (45.07%)	0.4217^G^
Alcohol consumption	6 (54.55%)	3 (42.86%)	33 (50%)	0.8953^G^
Smoking	4 (36.36%)	2 (28.57%)	21 (31.34%)	0.9350^G^
Injectable drugs	2 (18.18%)	0	4 (6.06%)	0.4200^G^
Inhalant drugs	2 (18.18%)	0	5 (7.58%)	0.4619^G^
Previous hepatitis	0	2 (28.57%)	10 (14.71%)	0.1722^G^

^A^ANOVA, one criterion; ^G^: *G* test

Similarly, the comparison among the three groups regarding clinical aspects also showed no statistically significant differences for the frequencies of the evaluated characteristics. In the comparison between necroinflammation scores and degrees of hepatic fibrosis among the three subgroups, no significant difference was observed for necroinflammation scores. However, there was a statistically significant difference between the subgroups for fibrosis scores (*p* = 0.0165). Individuals from the subgroup of ANA-positive patients with *RR* presented a significantly higher prevalence of higher degrees of hepatic fibrosis compared to the subgroup of ANA-negative patients (*G* test, *p* = 0.0042) (Table 6).

**Table 6:** Comparison of clinical data for ANA-positive *RR*-positive, ANA-positive *RR*-negative, and ANA-negative patients

Clinical characteristic	*RR*-positive *N* (%)	*RR*-negative *N* (%)	ANA-negative *N* (%)	*p-*value
Systemic arterial hypertension	6 (54.55%)	2 (28.57%)	24 (35.82%)	0.4629
Diabetes mellitus 2	2 (18.18%)	0	13 (19.4%)	0.2778
Hepatomegaly	0	0	11 (16.4%)	0.0864
Hepatic steatosis	2 (18.18%)	3 (60%)	11 (17.1%)	0.1243
Cirrhosis	4 (36.36%)	2 (28.57%)	9 (12.7%)	0.1716
Inflammatory activity^G^				
A0	0	1 (33.3%)	4 (4.59%)	
A1	6 (66.7%)	1 (33.3%)	27 (33.7%)	0.5442
A2	3 (33.3%)	1 (33.3%)	18 (26.5%)	
A3	0	0	4 (5.9%)	
Degree of fibrosis^G^				
F0	0	0	4 (7.55%)	
F1	0	2 (66.7%)	20 (37.73%)	
F2	3 (33.3%)	0	16 (30.18%)	0.0165
F3	3 (33.3%)	1 (33.3%)	12 (22.64%)	
F4	3 (33.3%)	0	1 (1.89%)	

^G^*G* test

In the comparison of the quantification of parameters of liver damage, liver function, leukocytes, platelets, and VL among ANA-positive patients with *RR*, ANA-positive patients with other patterns, and ANA-negative patients, the slight differences between these subgroups were not statistically significant (Table 7).

**Table 7:** Comparison of the quantification of laboratory tests for ANA-positive *RR*-positive, ANA-positive *RR*-negative, and ANA-negative patients

Marker	*RR*-positive	*RR*-negative	ANA-negative	*p-*value
AST (U/L)	101.23 ± 92.51	71.04 ± 44.87	65.85 ± 37.88	0.3779^k^
ALT (U/L)	106.6 ± 84.04	55.95 ± 28.63	77.59 ± 54.47	0.2301^k^
GGT (U/L)	110.43 ± 86.45	97.72 ± 82.19	95.1 ± 19.95	0.6711^k^
ALP (U/L)	121.65 ± 44.14	122.4 ± 64.38	119.13 ± 56.18	0.9841^A^
TB (mg/dL)	0.92 ± 0.63	1.8 ± 2.19	0.86 ± 0.47	0.6678^k^
DB (mg/dL)	0.36 ± 0.36	0.49 ± 0.54	0.27 ± 0.17	0.9792^k^
IB (mg/dL)	0.61 ± 0.31	1.32 ± 1.86	0.59 ± 0.38	0.5923^k^
TP (g/dL)	7.22 ± 0.55	7.63 ± 0.86	7.59 ± 1.03	0.6204^A^
Albumin (g/dL)	3.97 ± 0.53	4.27 ± 0.55	4.23 ± 0.65	0.3685^k^
Globulin (g/dL)	3.37 ± 0.87	3.35 ± 0.65	3.36 ± 1.04	0.9787^k^
PT (%)	84.74 ± 15.91	74.75 ± 18.93	83.32 ± 20.51	0.5493^A^
Platelets (× 10^3^)	157.8 ± 54.4	196.7 ± 104.86	205.75 ± 76.9	0.2211^k^
VL (log_10_)	5.64 ± 0.39	5.51 ± 0.27	5.49 ± 0.84	0.8064^k^

^K^Kruskal–Wallis test; ^A^ANOVA, one criterion

## Discussion

The prevalence of ANA in patients with chronic HCV infection in this study was 20.2%, which is significantly higher than the 2% prevalence found in healthy controls. This difference relative to the normal population is consistent with the data reported in previous studies [9, 19]. Moreover, in terms of percentage values, this prevalence is close to those found in other studies that reported frequencies between 19.3 and 21.3% [7, 20–23]. Even higher frequencies of ANA (between 32 and 42.6%) have been reported in patients with HCV [24–26].

The prevalence rates found in this study are well above the rates described in other studies (between 4.4% and 17.6%) [19, 27–32]. In Brazil, Narciso-Schiavon et al. [33] and Marconcini et al. [34] found prevalence rates of 9.4 and 7.6%, respectively.

Among the factors that may result in these differences, first, it must be taken into account that in most of these studies, analyses were performed on samples with a 1/40 titration, which, in theory, may increase the number of positive results due to a higher concentration of autoantibodies in the sample. Exceptions include the studies conducted by Daschakraborty et al. [22] and by Chrétien et al. [25], who analyzed samples diluted 1/80, and the studies conducted by Acay et al. [7], Łapiński et al. [23], and Kirdar et al. [31], who worked with samples diluted 1/100. Furthermore, there are differences between the substrates available in each HEp-2 cell kit, which can also generate differences in terms of pattern detection [35]. Another possibility stems from the association of fluorescence patterns with specific antibodies and differences in the susceptibility levels of different populations to develop one or another of these specific autoantibodies.

In general, the occurrence of ANA has a significantly closer association with female sex, older age, and the use of some classes of drugs [36–39]. In the present study, there was a slightly higher prevalence of ANA among female patients, although this difference was not significant. Data on the role of sex in the induction of ANA in the context of chronic hepatitis C are divergent: while some studies [9, 24, 32] found a relationship between ANA seroreactivity and female sex, others [25, 33, 34, 36] did not find such an association. Recently, direct-acting antivirals HCV clearance may interrupt chronic immune stimulation by removing the drive for autoantibody induction [40].

In addition, no association was found between the presence of ANA and any of the other epidemiological factors evaluated. Discordant data were found by Chen et al. [21] and by Hsieh et al. [24], who found a higher frequency of autoantibodies in older individuals. However, data similar to those in the present study were observed regarding the lack of an association of ANA with alcohol consumption [32, 33], age [34, 36], smoking [32], and the use of injectable drugs [25].

In the present study, it was not possible to associate the presence of ANA with any of the clinical characteristics evaluated in the patients, similar to the findings of other studies [25, 27, 33, 34]. However, Lenzi et al. [19] found a higher prevalence of ANA in patients with chronic hepatitis without cirrhosis than in those with cirrhosis, the opposite of the finding in the present study, although we did not observe a significant difference.

The possibility of ANA interference in liver damage and liver function assessment tests has been reported in several studies that have shown a relationship between the presence of these autoantibodies and elevated transaminases [21, 24, 25, 33], GGT [19, 25], and globulin [20, 23, 25] levels and the reduction in the number of platelets [21, 34] in ANA-positive patients, but not ANA-negative patients, with chronic HCV infection; however, there are studies that, similar to the present study, did not observe such associations [29, 34].

Moreover, as in the present study, most studies conducted by other authors report the absence of an association between the presence of ANA and VL [9, 31, 34]. However, a study by Hsieh et al. [26] reported a higher level of ANA in individuals with a lower VL. According to the authors, this difference may result from potentially associated genetic and environmental aspects.

The histopathological analysis performed in our study showed no difference in fibrosis scores between ANA-positive and ANA-negative patients, although we observed a higher prevalence of ANA in degrees F3 and F4. In this regard, the literature provides controversial data, with reports of an association of ANA with a greater liver disease severity [24, 25, 34] as well as reports of the absence of such an association [29, 31, 33].

In the context of genotypic variability of HCV, the analysis of subgroups of ANA-positive and ANA-negative patients showed no significant differences in the distributions of HCV genotypes 1 and 3 among these subgroups. These data are in agreement with the observations reported by some authors [31, 32, 34], although there are reports of an association of genotype 1 virus infection with the presence of ANA [23, 24, 30].

Additionally, in the present study, HCV subgenotype 1a was the most frequent among ANA-positive patients, although this difference relative to the subgroup of ANA-negative patients did not reach statistical significance. This finding suggests the possibility of an association between this subgenotype and the induction of ANA in patients with chronic HCV infection. However, the limited number of subgenotype analyses in this study and the lack of data in the literature on this type of analysis do not allow confirmation of this hypothesis.

Data from different studies show that in samples from HCV-positive patients, IIF analysis in HEp-2 cells shows a predominance of nuclear fluorescence patterns and an absence of cytoplasmic patterns. Nuclear patterns are mainly represented by speckled patterns, with frequencies varying between 36 and 90%, much higher than those found for the other commonly associated patterns, such as the homogeneous nuclear pattern and nucleolar patterns [19, 25–27, 33, 34]. However, in our study, there was a higher prevalence of cytoplasmic patterns, and among the nuclear patterns, there was an equal frequency of homogeneous nucleolar and speckled patterns.

The *RR* is usually associated with chronic HCV infection treated with a combination of pegylated IFN and ribavirin [30–43]. However, data from other authors indicate that the ribavirin-dependent mechanism is not essential for the induction of these autoantibodies because this ANA pattern can also be detected in individuals with systemic lupus erythematosus [44], in individuals with HBV infection [41], and in clinically healthy individuals [45]. In addition, this pattern can also be induced by other drugs, such as mycophenolic acid, azathioprine, methotrexate, and acyclovir [42, 46].

Existing data show frequencies of this pattern ranging from 14.1 to 37% in samples from individuals with chronic HCV infection [43, 47, 48], which are lower than the frequency (61.1%) found in the present study. These differences may result from the influence of host genetic aspects as well as from environmental factors, which may favor the formation of these autoantibodies in this region.

The epidemiological, clinical, laboratory, and molecular data did not differ among patients with *RR*, *RR*-negative patients, and ANA-negative patients. Recently, Assandri and Montanelli [49] showed the *RR* circulating autoantibodies in one patient with Primary Biliary Cholangitis, demonstrating that *RR* autoantibodies can also be present in autoimmune hepatitis case. Considering the *RR*-positive and *RR*-negative subgroups, some studies [41, 50, 51] did not find significant differences when considering patient age and sex. In addition, Stinton et al. [52] found no association of this ANA pattern with the use of injectable drugs. In terms of clinical characteristics, Covini et al. [47] and Da Silva Sacerdote et al. [51] found no association of this pattern with the occurrence of DM2 or the presence of cirrhosis. Similarly, Covini et al. [47] also found no association of this pattern with steatosis.

At the laboratory level, liver marker analyses performed in some studies [47, 51, 52] showed no association between the presence of *RR* and changes in biochemical tests. Molecular analyses [46, 47, 53] also did not show any association of this pattern with VL or with viral genotype.

In the present study, there was an association between the presence of *RR* and higher liver fibrosis scores, which differs from the observations reported by Climent et al. [48] and by Novembrino et al. [53], who found no similar association. This difference may be explained by the fact that, in those studies, the focus was only on patients undergoing treatment, while in our study, patients were selected using different inclusion criteria, such as not undergoing treatment at the time of data collection.

The small number of patients analyzed is a limitation of the present study. This limitation is due to the exclusion of patients from the study who had insufficient data and/or sample quantity. More generally, the discrepancies between the data available in the literature on the prevalence of ANA and its associations may be due to differences in dilutions, technical aspects, sensitivity of the laboratory methods, sample sizes, groups evaluated in each study, and population-related aspects, such as genetics and environment [9, 31].

## Conclusion

From a general perspective, the results of the present study confirm a potential for HCV to disrupt self-tolerance mechanisms and to induce ANA production. However, prospective studies and/or studies with larger sample sizes should help further elucidate the associations between the presence of ANA and different clinical-laboratory, molecular, and histopathological aspects of the disease, enabling a better understanding of the role of this marker in the context of chronic HCV infection. Additionally, further studies should be conducted to determine the role of the *RR* in the context of chronic hepatitis C to elucidate whether, in fact, the autoantibodies associated with this pattern play a role in the pathogenesis of liver disease and can be used as a monitoring biomarker of liver disease associated with this infection.

## Data Availability

The dataset used in the current study is available from the corresponding author upon reasonable request.

## Abbreviations

ALP: Alkaline phosphatase
ALT: Alanine aminotransferase
ANA: Antinuclear antibodies
AST: Aspartate aminotransferase
DB: Direct bilirubin
GGT: Gamma-glutamyl transferase
HBV: Hepatitis B virus
HCV: Hepatitis C virus
HIV: Human immunodeficiency virus
IB: Indirect bilirubin
IFN: Interferon
IIF: Indirect immunofluorescence
LACEN: Central Laboratory of the State of Pará
PT: Prothrombin time
RR: Rods and rings
TB: Total bilirubin
TP: Total protein
VL: Viral load
